# ZnT8 Deficiency Protects From APAP-Induced Acute Liver Injury by Reducing Oxidative Stress Through Upregulating Hepatic Zinc and Metallothioneins

**DOI:** 10.3389/fphar.2021.721471

**Published:** 2021-08-03

**Authors:** Wen Su, Mingji Feng, Yuan Liu, Rong Cao, Yiao Liu, Junyao Tang, Ke Pan, Rongfeng Lan, Zhuo Mao

**Affiliations:** ^1^ School of Basic Medical Sciences, Shenzhen University Medical Center, Shenzhen University Health Science Center, Shenzhen, China; ^2^ Department of Nephrology, The First Affiliated Hospital of Shenzhen University, Shenzhen, China; ^3^ Institute for Advanced Study, Shenzhen University, Shenzhen, China; ^4^ Department of Cell Biology and Medical Genetics, School of Basic Medical Sciences, Shenzhen University Health Science Center, Shenzhen, China

**Keywords:** ZnT8, acetaminophen, hepatotoxicity, metallothionein, oxidative stress

## Abstract

Zinc transporter 8 (ZnT8) is an important zinc transporter highly expressed in pancreatic islets. Deficiency of ZnT8 leads to a marked decrease in islet zinc, which is thought to prevent liver diseases associated with oxidative stress. Herein, we aimed to investigate whether loss of islet zinc affects the antioxidant capacity of the liver and acute drug-induced liver injury. To address this question, we treated ZnT8 knockout (KO) or wild-type control mice with 300 mg/ kg acetaminophen (APAP) or phosphate-buffered saline (PBS). Unexpectedly, we found that loss of ZnT8 in mice ameliorated APAP-induced injury and was accompanied by inhibition of c-Jun N-terminal kinase (JNK) activation, reduced hepatocyte death, and decreased serum levels of alanine aminotransferase (ALT) and aspartate aminotransferase (AST). An increase in hepatic glutathione (GSH) was observed, corresponding to a decrease in malondialdehyde (MDA) and 4-hydroxynonenal (4-HNE) levels. APAP-induced inflammation and glycogen depletion were alleviated. In contrast, no significant changes were observed in cytochrome P450 family 2 subfamily E member 1 (CYP2E1), the main enzyme responsible for drug metabolism. Elevated levels of hepatic zinc and metallothionein (MT) were also observed, which may contribute to the hepatoprotective effect in ZnT8 KO mice. Taken together, these results suggest that ZnT8 deficiency protects the liver from APAP toxicity by attenuating oxidative stress and promoting hepatocyte proliferation. This study provides new insights into the functions of ZnT8 and zinc as key mediators linking pancreatic and hepatic functions.

## Introduction

Zinc transporter 8 (ZnT8), encoded by the human SLC30A8 gene, is a zinc transporter closely associated with type 1 and type 2 diabetes (Barragán-Álvarez et al., 2021). ZnT8 is expressed almost exclusively in pancreatic β cells and is responsible for the uptake of zinc ions into insulin granules. Zinc ions in the pancreas are co-secreted with insulin into the portal vein and the liver to assist in primary insulin clearance. Previous studies in rodent models have shown that ZnT8 deficiency leads to a significant decrease in zinc levels in the pancreatic islets, resulting in degradation of insulin by the liver due to the lack of zinc (Tamaki et al., 2013). The pancreas is closely related to the liver in term of anatomy and function. Zinc may act as an important messenger mediating the pancreas-liver cross-talk. Therefore, we hypothesized that dysregulation of zinc homeostasis in ZnT8 knockout (KO) mice may contribute to altered liver function.

The liver is a vital multifunctional organ that secretes bile acids, regulates lipid and glucose hemostasis, and metabolizes drugs and xenobiotics (Trefts et al., 2017). Zinc is important for the oxidative status and metabolism of drug toxicity in the liver. (Prasad and Bao, 2019). Acetaminophen (APAP) overdose is the leading cause of drug-induced liver failure in the Western world (Lee, 2017). The mechanisms of APAP hepatotoxicity are complex and have been substantially investigated. Briefly, APAP is metabolized by cytochrome P450 enzymes to N-acetyl-p-benzoquinone imine (NAPQI), a highly reactive and toxic metabolite. NAPQI is detoxified by glutathione (GSH); however, if GSH is depleted, excess NAPQI can lead to toxicity and cell necrosis (Yan et al., 2018). Further, genetic predisposition may play a significant role in the sensitivity of individuals to drug-induced hepatotoxicity.

In this study, we aimed to investigate whether pancreatic-enriched ZnT8 affects hepatic drug toxicity. Interestingly, ZnT8-deficient mice were more resilient, rather than more sensitive, to APAP-induced liver injury. Both hepatic oxidative stress and inflammation were significantly reduced in the livers of ZnT8 KO mice. Further investigation showed that the zinc content in the liver of APAP-treated ZnT8 KO mice was significantly increased compared to wild-type control mice. Moreover, the expression levels of metallothioneins (MTs), proteins involved in metal detoxification, were substantially increased. These results suggest that genetic changes in the pancreas play an unexpected role in hepatic metabolism.

## Materials and Methods

### Animals

Eight-week-old male ZnT8 KO mice and wild-type control mice were placed at 20–25°C and maintained on a 12-h light/dark cycle. APAP was freshly dissolved in warm (55–60°C) distilled phosphate-buffered saline (PBS) and cooled to 37°C. Prior to APAP treatment, mice were fasted overnight and allowed to drink ad libitum. Mice were administered APAP intraperitoneally at a dose of 300 mg/ kg body weight, or PBS. Mice were sacrificed at various time points after injection. Serum and liver samples were harvested for analysis. All animal experiments were performed in accordance with the National Institute of Health Guide for the Care and Use of Laboratory Animals and were approved by the Scientific Investigation Board of Health Science Center of Shenzhen University (Shenzhen, Guangdong, China).

### Antibodies and Chemicals

CYP2E, CYP4A, CDK2, CDK4, and MT antibodies were purchased from Abcam (Cambridge, MA, United States). Phospho-JNK (pJNK), JNK, and F4/80 antibodies were from Cell Signaling Technology, Inc. (Beverly, MA, United States). PCNA, GAPDH, and tubulin antibodies were from Proteintech Group (Wuhan, China). APAP was obtained from Sigma-Aldrich (St Louis, MO, United States).

### Biochemical Analysis

Liver GSH assay kit was purchased from Oxford Biomedical Research Company (Rochester Hills, MI). Malondialdehyde (MDA) assay kit, superoxide dismutase (SOD) kit, serum alanine aminotransferase (ALT) assay kit, and aspartate aminotransferase (AST) assay kits were purchased from Nanjing Jiancheng Bioengineering Institute (Nanjing, China).

### Histological and Immunohistochemical Staining

Liver samples were fixed in 4% paraformaldehyde and embedded in paraffin, and then section into 4–6 μm thick slices. Sections were stained with hematoxylin and eosin (H and E) to analyze hepatic pathological damage. Images were obtained using a Nikon Eclipse Ti microscope. Immunohistochemical staining was performed as previously described (Mao et al., 2019).

### Oil Red O, Periodic Acid-Schiff, and TUNEL Staining

Frozen liver sections were washed once in PBS and fixed with 4% paraformaldehyde (PFA) in PBS for 15 min at room temperature, followed by three washes in PBS. Sections were incubated in 60% isopropanol and then stained with filtered Oil Red O solution (1.5 mg/ ml) for 30 min and rinsed twice with distilled water. PAS staining of glycogen was performed using a commercial kit according to the manufacturer’s instructions (Solarbio, Beijing, China). TUNEL assays were performed according to the *In Situ* Cell Death Detection Kit, Fluorescein (Roche, Basel, Switzerland).

### Timm’s Staining

Liver tissue sections fixed in 4% (w/v) PFA/PBS were immersed in 0.1% Na_2_S in 0.1 M PBS for 1 h, 3% glutaraldehyde in 0.15 M PBS for 1 h, and then 0.1% Na_2_S in 0.1 M PBS for 1 h. The sections were then incubated for 60 min in developing solution (30 ml 50% gum arabic, 5 ml 2 M citrate buffer pH 3.7, 15 ml 5.67% hydroquinone, and 0.25 ml 17% AgNO_3_) protected from light and stirred gently. The slides were then rinsed several times in water and observed under a stereomicroscope.

### Measurement of Zn^2 +^ Using Flame Atomic Absorption Spectroscopy

Liver tissues were lyophilized and weighed prior to zinc concentration measurement. Tissue and serum were then digested with concentrated nitric acid (70%, Fisher Scientific, Waltham, MA) and heated in an auto-regulated heating block at 110°C for 48 h. All completely digested samples were diluted, and Zn^2+^ concentrations were quantified using a Perkin-Elmer Analyst 800 atomic absorption spectrometer. The recoveries of Zn^2+^ in standard reference material ranged between 95 and 110%.

### Western Blotting, RNA Extraction and qRT-PCR

Western blotting was performed with 40 μg of protein lysate as described previously (Mao et al., 2021). Total RNA was extracted from mouse tissues, and cDNA synthesis and SYBR green gene expression assays were performed as previously described (Mao et al., 2021).

### Statistical Analysis

All data are presented as mean ± standard error of the mean (SEM). Statistical differences between paired groups were measured by the unpaired Student’s t-test or two-way analysis of variance (ANOVA) followed by Bonferroni’s multiple comparisons test. All statistical analyses were performed using Prism software (GraphPad 8.0). *p*-values < 0.05 were considered statistically significant.

## Results

### ZnT8 KO Mice Are Protected From APAP-Induced Liver Injury

**FIGURE 1:**
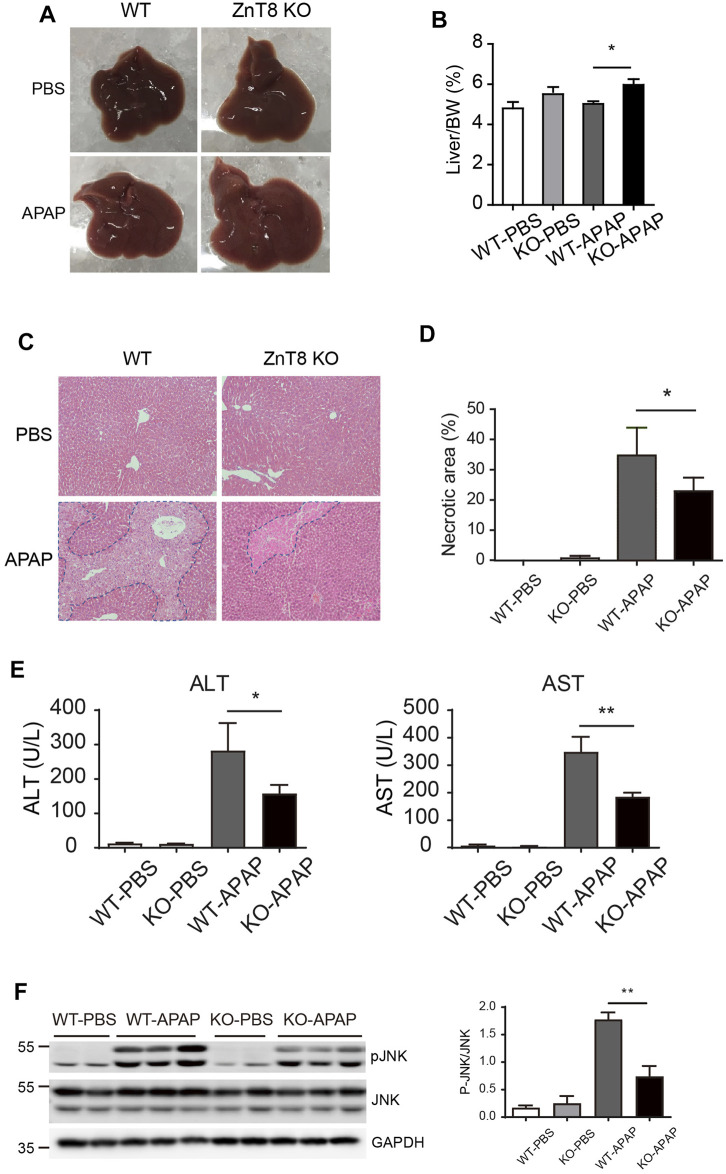
ZnT8 deficiency ameliorates APAP-induced liver injury. Mice were treated with 300 mg/ kg APAP and sacrificed 24 h after APAP treatment. **(A)** Gross morphology of the livers. **(B)** Liver weights. **(C)** Representative H and E stained images. **(D)** Quantification of necrotic area. **(E)** Serum ALT and AST activities. **(F)** Immunoblotting and quantification of pJNK and total JNK in the liver. Data are represented as mean ± SEM. *, *p* < 0.05, **, *p* < 0.01 by the two-way ANOVA and post hoc Bonferroni’s multiple comparison test.

To investigate whether ZnT8 plays a role in drug-induced hepatotoxicity, we injected APAP intraperitoneally into ZnT8 KO and wild-type mice. The mice were sacrificed and analyzed 24 h after injection. The liver mass, expressed as a percentage of total body weight, was heavier in ZnT8 KO mice than in wild-type mice (Figures 1A,B). Histological analysis showed that ZnT8 KO mice had less typical bridging necrosis within the centrilobular region and smaller areas of necrosis compared with wild-type controls (Figure 1C). Quantification of APAP-induced liver damage after 24 h indicated that approximately 22% of the liver was necrotic in ZnT8 KO mice compared to approximately 33% in wild-type mice (Figure 1D). APAP treatment resulted in wild-type mice with a significant increase in liver injury markers ALT and AST after 24 h, while ZnT8 KO mice showed a lesser increase (Figure 1E).

Activation of JNK, a marker of APAP-induced liver injury, is activated by phosphorylation of JNK protein in response to APAP treatment. The level of activation correlates with the degree of injury. We measured total and activated (phosphorylated) JNK levels by western blotting. Treatment of wild-type mice with APAP for 6 h strongly induced activation of JNK in the liver, whereas ZnT8 KO mice were resistant to APAP-stimulated JNK activation in the liver (Figure 1F). These results suggest that the lack of ZnT8 protects mice from APAP-induced hepatotoxicity.

### Oxidative Stress Was Reduced in APAP-Treated ZnT8 KO Mice

**FIGURE 2:**
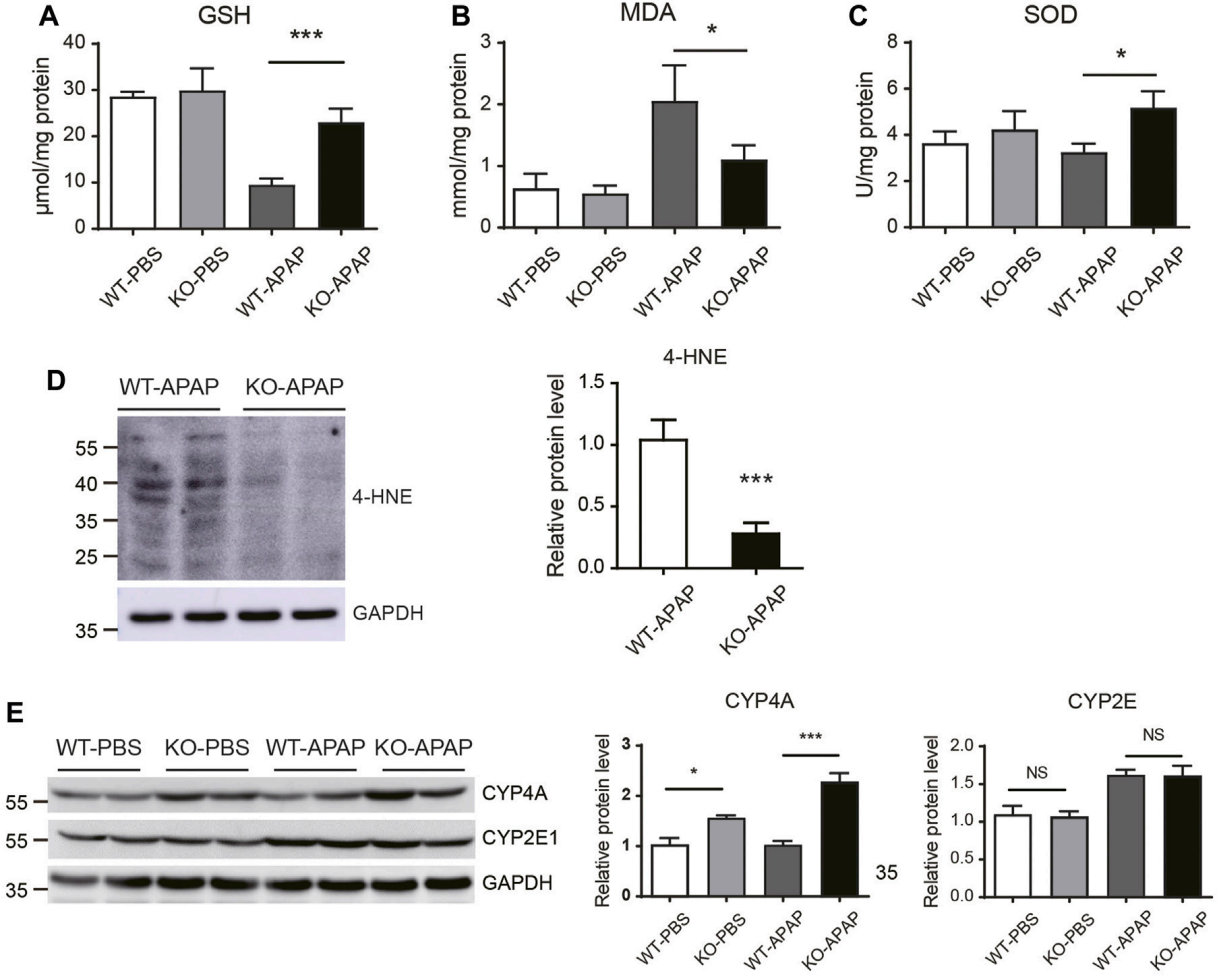
Oxidative stress is reduced in the liver of APAP-treated ZnT8 KO mice. Hepatic levels of GSH **(A)**, MDA **(B)**, and SOD **(C)**. **(D)** Immunoblotting and quantification of 4-HNE in the liver. **(E)** Immunoblotting and quantification of CYP4A and CYP2E1 in liver. GAPDH was used as an internal control. Data are represented as mean ± SEM. *, *p* < 0.05, ***, *p* < 0.001 by the one-way ANOVA test or unpaired Student’s *t*-test. NS, not significant.

GSH depletion and oxidative stress have been shown to play a key role in APAP-induced hepatotoxicity. APAP is bioactivated by cytochrome P450 to form NAPQI, which then binds to cellular proteins, forming APAP-protein adducts and leading to GSH depletion and oxidative stress (Yan et al., 2018). To investigate whether the attenuated APAP-induced liver injury in ZnT8 KO mice is the result of altered APAP metabolism, we examined GSH consumption and APAP metabolism. Hepatocellular GSH loss was reduced in ZnT8 KO mice compared to wild-type mice 6 h after APAP treatment (Figure 2A), suggesting that ZnT8 deficiency has a lesser bioactivating effect on APAP and consumes less GSH.

Lipid peroxidation is a key indicator of APAP-mediated oxidative damage in the liver (Saito et al., 2010b). The lipid peroxidation product MDA was decreased in APAP-treated ZnT8 KO livers compared to control livers. Consistently, the antioxidant enzyme SOD was increased in ZnT8 KO livers (Figures 2B,C). Another marker of lipid peroxidation is 4-Hydroxynonenal (4-HNE). Consistently, APAP induced a significant decrease in 4-HNE levels in the livers of ZnT8 KO mice (Figure 2D).

Cytochrome P450 (CYP) enzymes are the predominant enzymes involved in hepatic drug metabolism, and CYP2E1 is the major enzyme responsible for metabolizing APAP (Tracy et al., 2016). We examined CYP2E1 protein expression but detected no difference between ZnT8 KO mice and control mice (Figure 2E). In contrast, CYP4A, another important oxidative metabolism enzyme, was markedly increased, which is consistent with our recent study showing that CYP4A has a protective effect in bile duct ligation (BDL) induced liver injury (Figure 2E) (Li et al., 2021). We also compared the gene expression levels of other major APAP-metabolizing enzymes and transporters in the livers. However, we did not detect any significant changes in these genes (Supplementary Figure 1). Together, these results suggest that ZnT8 deficiency prevents APAP-induced liver injury by inducing CYP4A, reducing GSH depletion, and suppressing lipid peroxidation.

### Hepatic Proliferation Is Accelerated in ZnT8 KO Mice

**FIGURE 3:**
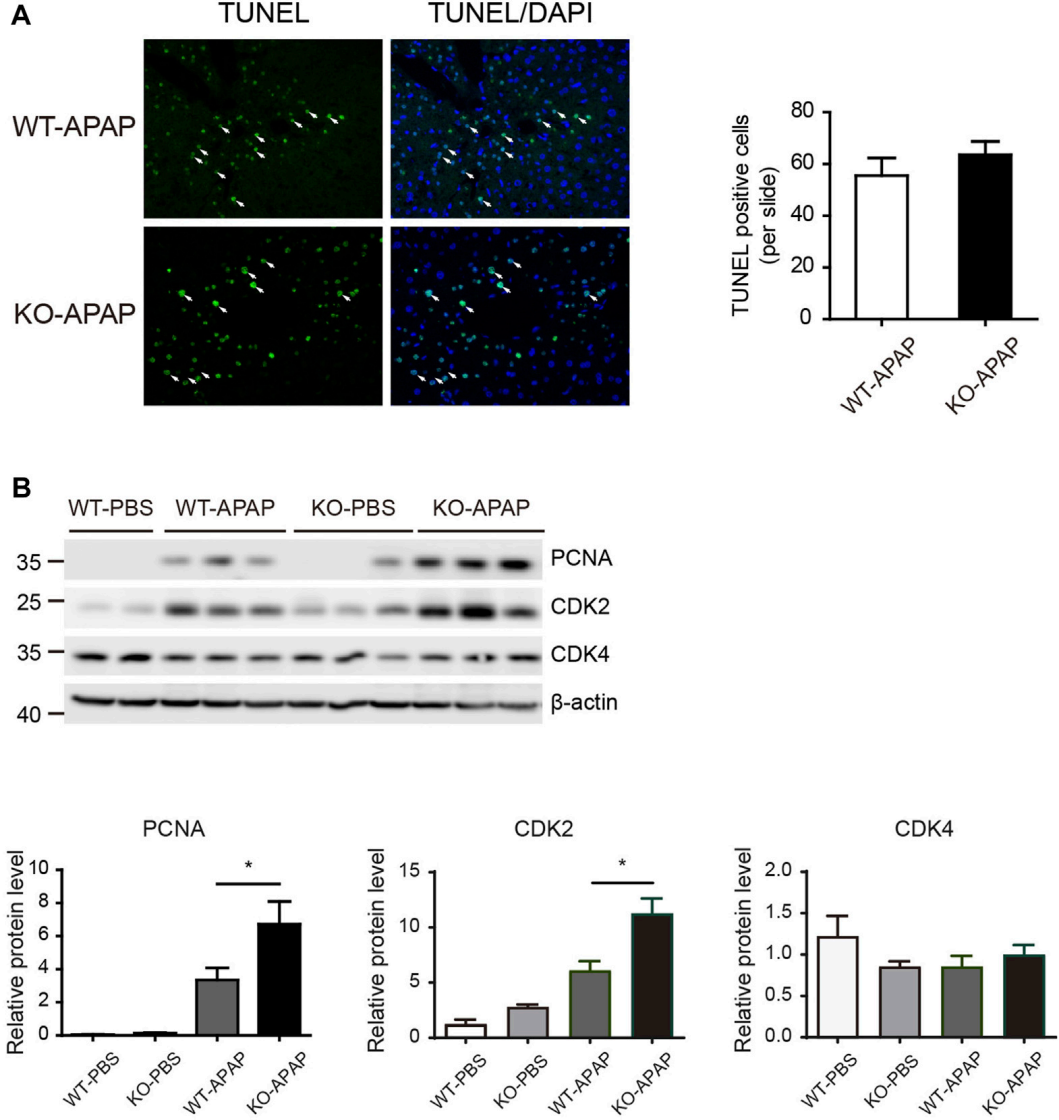
Increased proliferation in APAP-treated ZnT8 KO livers. **(A)** Representative TUNEL staining images and quantification of TUNEL-positive cells. **(B)** Immunoblotting and quantification of PCNA, CDK2, and CDK4 in liver. β-tubulin was used as an internal control. Data are expressed as mean ± SEM. *, *p* < 0.05 by the one-way ANOVA test or unpaired Student’s *t*-test. NS, not significant.

APAP-induced hepatotoxicity also depends on the balance between hepatocyte death and regeneration. The increased liver weight in APAP-treated ZnT8 KO mice compared to control wild-type mice (Figure 1B) suggests that this process may also be affected. We labeled apoptotic cells with the TUNEL assay, but there were no differences between ZnT8 KO mice and wild-type mice (Figure 3A). Proliferating cell nuclear antigen (PCNA) is a marker of cells with proliferative potential, while cyclin-dependent kinases (CDKs) are important regulators of cell cycle control. Hepatic PCNA and CDK2 protein levels were significantly increased in APAP-treated ZnT8 KO mice (Figure 3B). These results suggest that the increase in hepatocyte proliferation may ameliorate APAP-induced liver injury in ZnT8 KO mice.

### Inflammation Is Reduced in ZnT8 KO Mice

**FIGURE 4:**
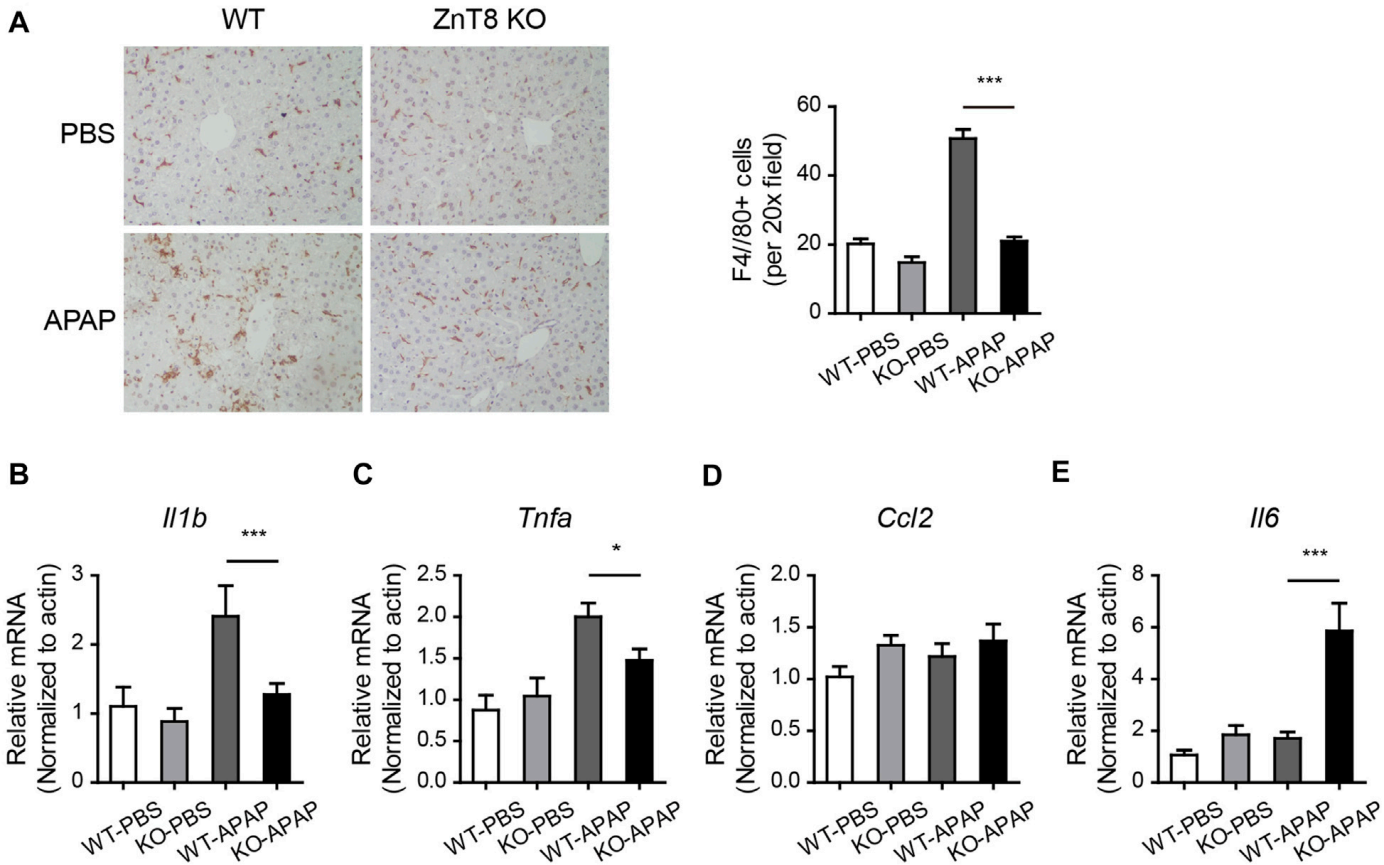
Reduced inflammation in APAP-treated ZnT8 KO livers. **(A)** Representative IHC staining images and quantitative analysis of F4/80 in the liver. **(B–E)** Relative transcript levels of liver inflammatory genes Il1b **(B)**, Tnfa **(C)**, Ccl2 **(D)**, and Il6 **(E)**. Data are represented as mean ± SEM. *, *p* < 0.05, ***, *p* < 0.001. By tested one-way ANOVA.

Altered inflammatory responses and cytokine levels were observed in APAP-induced injury. Macrophage marker F4/80 staining showed a significant reduction in macrophage infiltration in ZnT8 KO mice, suggesting an attenuated inflammatory state (Figure 4A). Consistently, the mRNA levels of the liver inflammatory markers interleukin 1 beta (Il1b) and tumor necrosis factor alpha (Tnfa) were also significantly reduced in ZnT8 KO mice (Figures 4B–D). Interestingly, mRNA expression of interleukin-6 (Il6) was increased in ZnT8 KO mice (Figure 4E). IL6 is known to play an important role in liver regeneration and acute liver injury (Gewiese-Rabsch et al., 2010; Schmidt-Arras and Rose-John, 2016), and IL-6 trans-signaling also significantly affects glycogen depletion in liver injury (Gewiese-Rabsch et al., 2010). Notably, APAP treatment, even as short as 6 h, causes glycogen depletion. We previously showed that ZnT8 deficient mice have more glycogen stored in the liver than wild-type mice (Mao et al., 2019). Here, ZnT8 KO mice also had larger glycogen stores than wild-type mice following APAP treatment (Supplementary Figure 2). These findings suggest that the inflammatory status and glycogen metabolism of ZnT8 KO mice are altered after APAP-induced liver damage.

### The Level of Hepatic Zinc Is Increased in ZnT8 KO Mice

**FIGURE 5:**
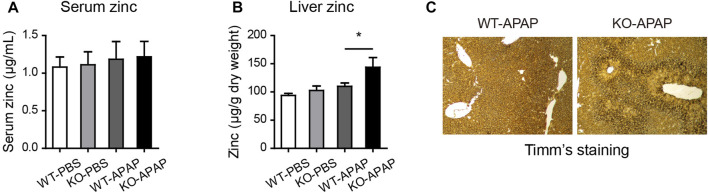
Elevated zinc levels in the liver of ZnT8 KO mice. **(A)** Serum zinc levels. **(B)** Liver zinc levels. **(C)** Representative images of Timm’s staining of the liver. Data are represented as mean ± SEM. *, *p* < 0.05 as tested by one-way ANOVA.

Zinc levels are associated with hepatic stress and inflammation status (Prasad and Bao, 2019). We analyzed liver and serum zinc levels using FAAS. There was an increase in liver zinc levels but no difference in serum zinc levels after APAP treatment (Figures 5A,B). Timm’s sulfide silver staining has been used to visualize various metals in the brain and other tissues. To confirm the observed zinc levels, we stained the liver tissue using Timm’s staining. Consistently, we found that the zinc levels, shown by brown signals, were remarkably increased in the centrilobular area, which correlated with the necrotic area of H&E staining (Figure 5C).

### The mRNA Expression Levels of Zinc Transporters Are Changed and MTs Is Increased in ZnT8 KO Mice

**FIGURE 6:**
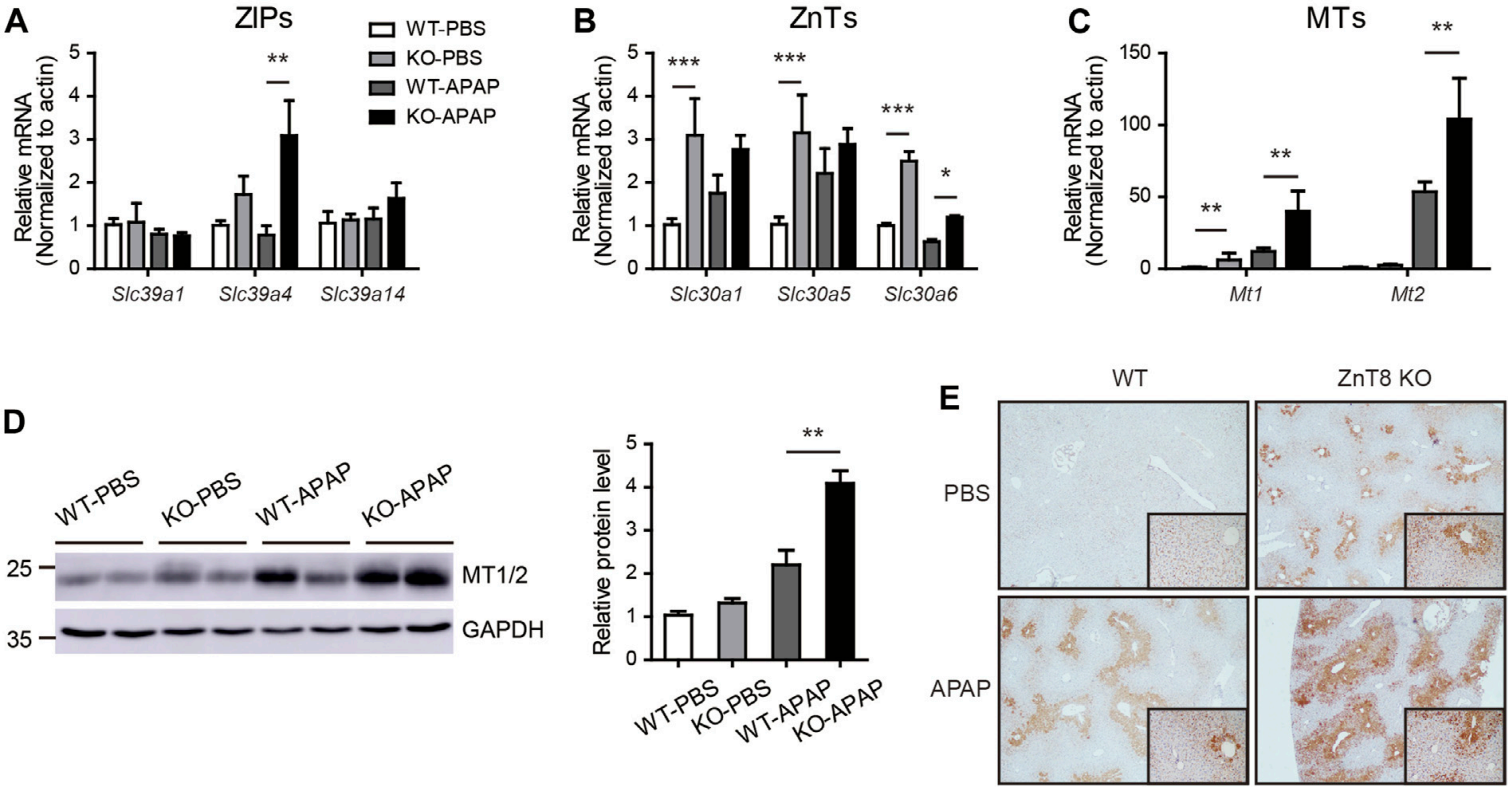
Altered mRNA expression profile of zinc transporters and increased MTs in ZnT8 KO mice livers. **(A–C)** The mRNA expression levels of ZIP **(A)**, ZnT **(B)**, and MT genes in the liver. **(D)** Immunoblotting and quantification of MT1/2 in liver. GAPDH was used as an internal control. **(E)** Representative immunohistochemical staining of MT1/2 proteins in liver. Data are represented as mean ± SEM, and analyzed by the one-way ANOVA test.

Zinc homeostasis is balanced mainly by two major zinc transporter families, the zinc influx ZIP (SLC39) proteins and the efflux ZnT (SLC30) proteins (Bafaro et al., 2017). We analyzed the mRNA expression levels of ZIPs and ZnTs in the liver. Under basal conditions, most ZIPs, including Slc39a4, Slc39a7, Slc39a11, and Slc39a13, were mildly induced in ZnT8 KO mice. Several ZnTs, including Slc30a1, Slc30a5, and Slc30a6, were induced in ZnT8 KO mice, indicating that zinc metabolism was more active in the livers of ZnT8 KO mice (Figures 6A,B, and Supplementary Figure 3). Following APAP treatment, *Slc39a4* and *Slc39a14* were the most highly upregulated zinc uptake transporters in the livers of APAP-treated ZnT8 KO mice, whereas the expression levels of most ZnTs were unchanged except for a slight increase in Slc39a6 levels (Figures 6A,B). These results suggest that ZIP4 and ZIP14 may contribute to the elevated zinc levels in the livers of ZnT8 KO mice.

Zinc levels are also regulated by a group of buffer proteins called MTs, which directly scavenge reactive oxygen species (ROS) and interact with GSH to enhance SOD activity (Formigari et al., 2007; Chiaverini and De Ley, 2010). Zinc may protect tissues from oxidative stress by inducing the expression of MTs. Indeed, in the liver of ZnT8 KO mice, both mRNA and protein levels of MTs were significantly increased (Figures 6C,D). Immunostaining showed that after APAP treatment, MTs were mainly enriched around the pericentral venous area, correlating with the positively stained zinc area. We also noted that hepatic MT levels were significantly elevated in both basal and APAP-treated livers of ZnT8 KO mice (Figure 6E). These results suggest that elevated hepatic zinc and MTs in ZnT8 KO mice may confer protection against oxidative stress and hepatic injury.

## Discussion

**FIGURE 7:**
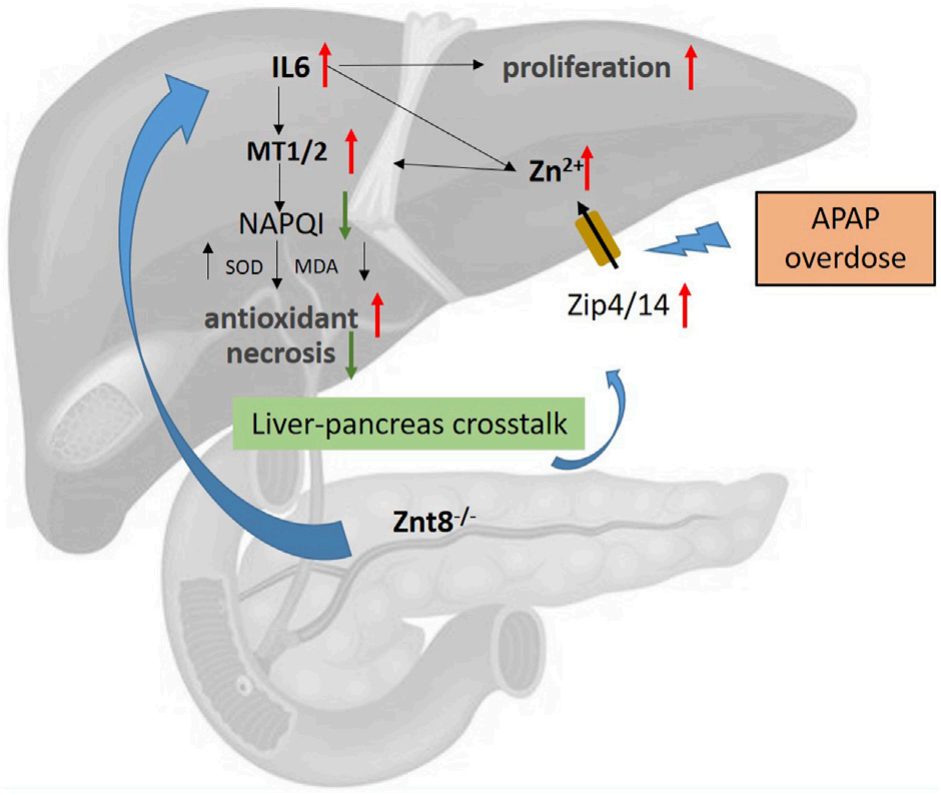
Potential mechanism of ZnT8 KO protection against APAP-induced liver injury. Zip4 and Zip14 are localized to the plasma membrane of hepatocytes and are dramatically upregulated after APAP treatment. ZnT8 KO mice displayed higher levels of hepatic IL6, Zip4, and Zip14 expression than wild-type mice after APAP overdose, accompanied by increased zinc uptake. Accumulation of hepatic Zn^2+^ and IL6 upregulates the antioxidant proteins MT1/2. Accumulation of MT1/2 attenuates APAP-induced inflammation, ROS, and necrosis. Zip4 and Zip14 may play a major role in the mechanisms responsible for the protective effects of ZnT8 KO mice. In addition, higher levels of IL6 also lead to enhanced hepatocyte proliferation in ZnT8 KO mice.

In this study, we initially hypothesized that ZnT8-deficient mice contain lower levels of pancreatic zinc than wild-type mice, which may deteriorate the antioxidant and detoxification functions of the liver. Unexpectedly, we observed ameliorated hepatic injury in APAP-treated ZnT8 KO mice. Mice were injected intraperitoneally with APAP and were sacrificed to examine the liver injury. ZnT8 KO mice exhibited decreased serum levels of hepatic enzymes ALT and AST. Histopathological examination revealed a significant decrease in the hepatocellular necrosis area in injured livers of ZnT8 KO mice. Consistently, oxidative stress was reduced in ZnT8 KO mice, and an increase in hepatic GSH was observed, along with a decreased in the levels of MDA and 4-HNE. No significant changes were observed in CYP2E1, the main enzyme responsible for drug biotransformation. APAP-induced inflammation and glycogen depletion were also alleviated. The increased levels of hepatic zinc and MTs may contribute to the hepatoprotective effect of ZnT8 KO mice. Taken together, our findings suggest that ZnT8 KO mice showed an unexpected improvement in antioxidant activity against APAP hepatotoxicity (Figure 7).

The initial aim of our study was to investigate whether alterations in pancreatic genetics could affect liver function. ZnT8, encoded by the SLC30A8 gene, is a pancreatic-enriched zinc transporter primarily responsible for transporting zinc into insulin granules. Genetic ZnT8 polymorphisms have been associated with defective β-cell zinc levels and diabetes mellitus (Sladek et al., 2007; Rutter and Chimienti, 2015). Previous studies have shown that zinc, which is secreted in concert with insulin, suppresses hepatic insulin clearance. In the absence of ZnT8, a substantial amount of insulin is degraded in the liver, resulting in low peripheral insulin levels (Tamaki et al., 2013). Zinc is known to be an important antioxidant that protects the liver from drug toxicity. Therefore, we hypothesized that altered zinc flow from the pancreas to the liver might affect hepatic injury associated with oxidative stress.

In Western countries, APAP-induced liver injury remains the leading cause of drug-induced liver injury (Lee, 2017). The pathogenesis of APAP hepatotoxicity is complex. Briefly, APAP is metabolized in the liver by CYP2E1 to form NAPQI, which is then conjugated and detoxified with GSH. Upon exhausting hepatic GSH, excess NAPQI eventually leads to mitochondrial damage and hepatocyte death (Yan et al., 2018).

Under normal conditions, zinc levels in the liver are relatively low. Zinc plays an important role in hepatic antioxidant and drug-metabolizing activities by preventing the formation of toxic cellular ROS and mitochondrial stress. Mechanisms of zinc in hepatic drug metabolism include: 1) direct binding of zinc to drugs; 2) zinc as a cofactor for most CYP450 family members; 3) zinc-induces the expression of MTs (Prasad and Bao, 2019). MTs are a family of low molecular mass, cysteine-rich metal-binding proteins, which buffer free zinc in the cytoplasm and protect it against toxic and oxidative stress-inducing effects. MTs are protective against APAP-induced liver injury through scavenging NAPQI after GSH depletion and preventing covalent binding to cellular proteins (Saito et al., 2010a). The expression of MTs is highly regulated by zinc levels, oxidative stress, inflammation, or cytokines such as IL6 (Babula et al., 2012). In our study, we unexpectedly found that basal liver and serum zinc levels were unaltered in ZnT8 KO mice. However, acute treatment with APAP in ZnT8 KO mice significantly increased hepatic zinc and MT1/2 protein levels. The protective effect of zinc and MTs against APAP injury or acute liver injury has been well demonstrated in previous reports (Chen et al., 2001; Saito et al., 2010a; Babula et al., 2012). Since our mouse model is deficient in a zinc transporter, we hypothesize that changes in zinc content and distribution are responsible for the protective effect against the acute liver injury. The lack of islet zinc flux may sensitize the liver to increase the expression of zinc transporters. When stimulated, the liver transports more zinc and defends against oxidative stress. Serum zinc levels were unaltered in ZnT8 KO mice. Therefore, it is unlikely that systemic zinc status affects the expression of hepatic MTs. The most likely alteration is zinc transport in the portal vein from the pancreas.

CYP2E1 is thought to be the major CYP responsible for NAPQI metabolism; however, no change in CYP2E1 expression was observed in ZnT8 KO mice. We hypothesized that the main protective mechanism occurred via zinc/MT signaling. CYP4A was upregulated under basal conditions and after APAP treatment in ZnT8 KO mice. In our previous study, we showed that ZnT8 KO mice exhibited a significant increase in hepatic lipid accumulation (Mao et al., 2019). We also noticed that several PPARα targeting genes were upregulated in the livers of ZnT8 KO mice, suggesting an activated PPAR signaling pathway. CYP4A is under the transcriptional control of PPARα in the liver. Therefore, one would suspect that enhanced PPARα activity is responsible for the increased CYP4A expression at the basal level. In addition, our recent paper showed that CYP4A exerts a protective effect in BDL-induced inflammation and liver injury, supporting the possibility that the increased CYP4A in the ZnT8 KO mice in this study may be participate in the attenuation of APAP-induced liver injury (Li et al., 2021).

In this study, we observed an increase in liver weight following APAP treatment, suggesting increased proliferation. The liver has a strong regenerative capacity after injury. We observed a significant increase in the number of proliferating hepatocytes in the transition zone between necrotic and healthy cells. PCNA and the cell cycle protein CDK2 were also elevated in ZnT8 KO mice livers. Previous reports have suggested that IL6 can make hepatocytes more responsive to growth factors and encourage proliferation (Kovalovich et al., 2000; Schmidt-Arras and Rose-John, 2016). In addition, IL6 is important for hepatocyte regeneration after APAP overdose in mice (James et al., 2003). Therefore, we speculate that IL6 may contribute to the increased proliferation in our ZnT8 KO model. Furthermore, IL6 induces hepatic ZIP14 expression and zinc accumulation (Liuzzi et al., 2005). In this study, Tnfa expression was reduced in ZnT8 KO mice compared to Il6, which may be a reflection of attenuated TNF-α/NFκB signaling and reduced inflammation. Increased IL6 levels and hyperactive IL6 signaling in ZnT8 KO livers may contribute to the increased hepatocyte proliferation, which may also attenuate APAP-induced liver injury by increasing the capacity of liver repair.

ZnT8 is expressed almost exclusively in pancreatic islets and is not found in the liver. ZnT8 deficiency stimulates rescue factors or reduces/inhibits pro-injury factors in an, as yet, unknown manner to maintain hepatocyte viability and reduce liver injury. We recently found that ZnT8 is also expressed in enteroendocrine cells and affects the intestinal microenvironment and microbiota (Mao et al., 2019). These factors may also influence the liver status and xenobiotic metabolism. In the present study, it was not possible to distinguish whether pancreatic or intestinal alterations affecting ZnT8 KO mice resulted in effects on the liver. Tissue-specific ZnT8 KO mice would be an option to address this question.

Interestingly, studies in humans have shown that ZnT8 loss-of-function mutants are associated with increased insulin secretion capacity and lower risk of developing type 2 diabetes (Flannick et al., 2014; Dwivedi et al., 2019). In this study, we also observed that ZnT8 deficiency protects against hepatic drug injury. Therefore, ZnT8 remains an attractive target for antidiabetic therapy, which may also confer antioxidant protection.

In conclusion, we have demonstrated that mice deficient in ZnT8 partially prevent APAP-induced liver injury. The resistance to APAP-induced liver injury is likely due to compensatory changes in zinc transporter expression, increased zinc accumulation, and increased expression of MTs.

## Data Availability

The raw data supporting the conclusions of this article will be made available by the authors, without undue reservation.
