# Supplementary material for: ZnT8 Deficiency Protects From APAP-Induced Acute Liver Injury by Reducing Oxidative Stress Through Upregulating Hepatic Zinc and Metallothioneins
Source: Front Pharmacol. 2021 Aug 3;12:721471. doi: 10.3389/fphar.2021.721471 (PMC8369884; doi:10.3389/fphar.2021.721471)
Supplement: Supplementary file 1 [file DataSheet1.PDF]

Supplemental figure legends

Figure S1. The expression profiles of APAP metabolism related genes.

(A-F) The mRNA expression levels of *Cyp1a1* (A), *Cyp1a2* (B), *Abcb4* (C), *Ugt1a1* (D), *Ugt1a6a* (E) and *Ugt1a6b* (F) in the liver. Data are represented as mean ± SEM. *P < 0.05, **P < 0.01, ***P < 0.001 by two-way ANOVA test. NS, not significant.

Figure S2. APAP-induced decrease of hepatic glycogen content in ZnT8 knockout livers was reduced.

Representative images and quantification of PAS staining in the liver. Data are represented as mean ± SEM. ***P < 0.001 by two-way ANOVA test.

Figure S3. Hepatic zinc transporter expression profiles in ZnT8 group mice.

(A-B) The mRNA expression levels of ZIP (A) and ZnT (B) proteins in the livers of ZnT8 ko and wild-type mice under the basal condition. Data are represented as mean ± SEM. **P < 0.01 by one-way ANOVA test.

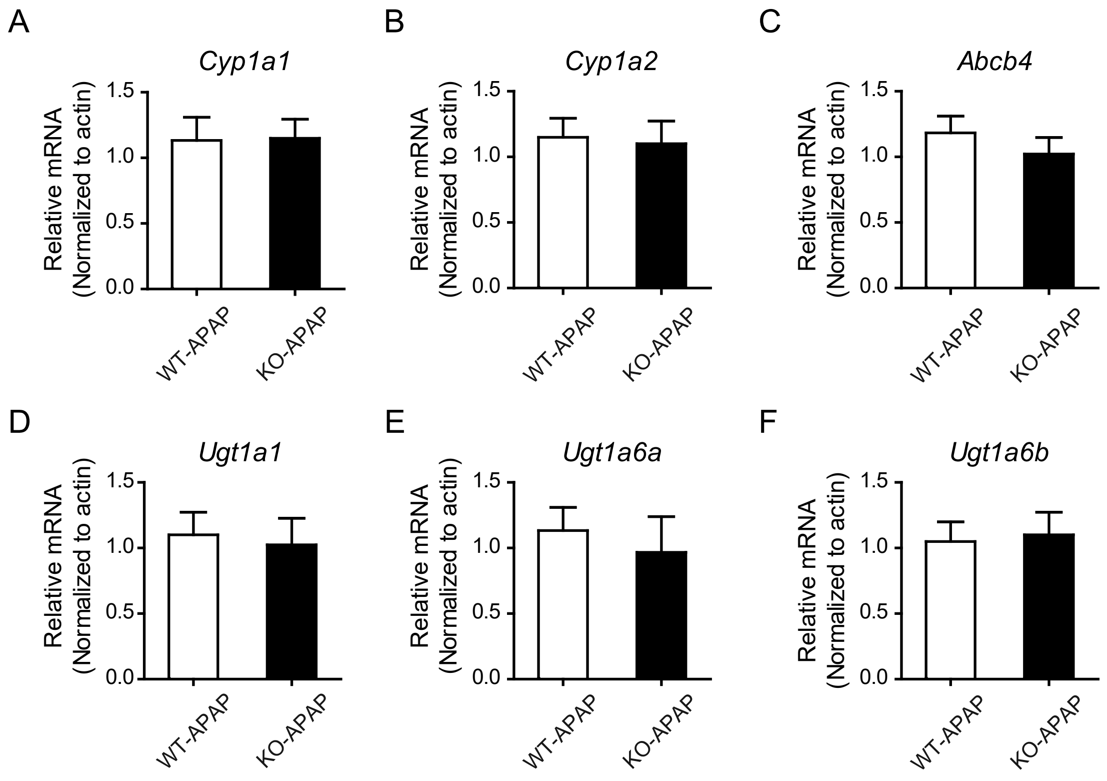

Figure S1. The expression profiles of APAP metabolism related genes

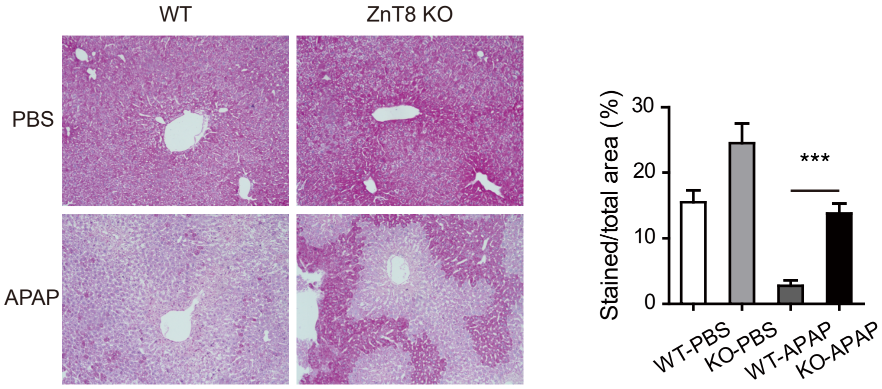

Figure S2. APAP-induced decrease of hepatic glycogen content was reduced in ZnT8 knockout livers.

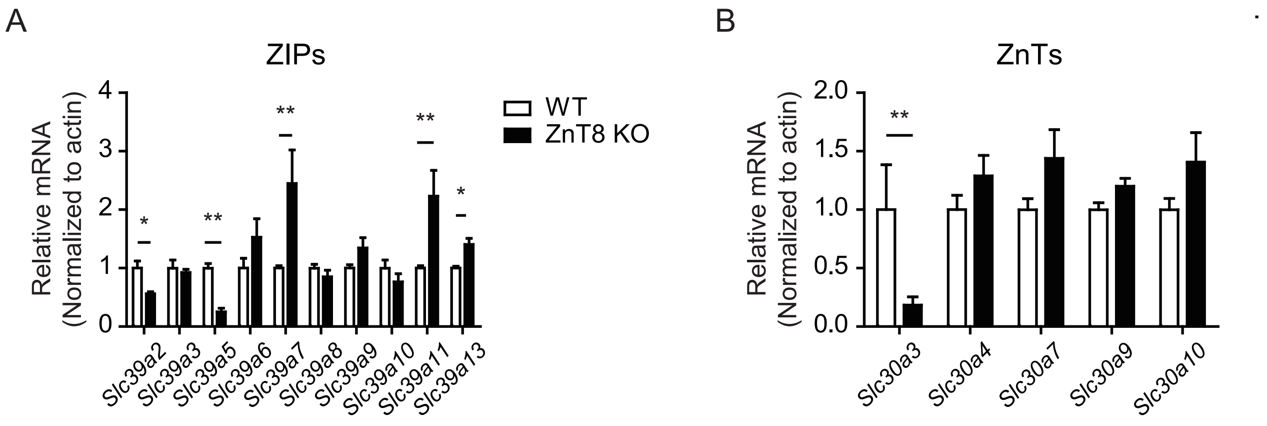

Figure S3. Hepatic zinc transporter expression profiles in ZnT8 group mice

Supplementary Table 1. Primers used for real-time quantitative PCR.

Gene	5' Sequence	3' Sequence
Abcb4	CAGCGAGAAACGGAACAGCA	TCAGAGTATCGGAACAGTGTCA
Ccl2	TTAAAAACCTGGATCGGAACCAA	GCATTAGCTTCAGATTTACGGGT
Cd36	ATGGGCTGTGATCGGAACTG	GTCTTCCCAATAAGCATGTCTCC
Cpt1a	GGGGTACCTATCCATCGTGAGCAGCYGG	CCGCTCGAGCCGGGATCCCCCAGATCC
Cyp1a1	GACCCTTACAAGTATTTGGTCGT	GGTATCCAGAGCCAGTAACCT
Cyp1a2	AGTACATCTCCTTAGCCCCAG	GGTCCGGGTGGATTCTTCAG
Fasn	GGAGGTGGTGATAGCCGGTAT	TGGGTAATCCATAGAGCCCAG
Gapdh	TGGCAAAGTGGAGATTGTTGC	AAGATGGTGATGGGCTTCCCG
Il1b	TCTTTGAAGTTGACGGACCC	TGAGTGATACTGCCTGCCTG
Il6	TAGTCCTTCCTACCCCAATTTCC	TTGGTCCTTAGCCACTCCTTC
Mt1	GCTGTGCCTGATGTGACGAA	AGGAAGACGCTGGGTTGGT
Mt2	GCCTGCAAATGCAAACAATGC	AGCTGCACTTGTCGGAAGC
Pgc1a	TCTGAGTCTGTATGGAGTGACTA	CCAAGTCGTTCACATCTAGTTCA
Scd1	GGGGTACCGAAGCTCCTGAAGTCTACAGTGG	CCGCTCGAGTTGGAAACCTGCCCTCCTGA
Slc30a1	CACGACTTACCCATTGCTCAAG	CTTTCACCAAGTGTTTGATATCGATT
Slc30a5	TGGACCACTAAGGACCTTGCT	CAGCCCCTCTTGTCTTTGC
Slc30a6	ATGGGGACGATTCATCTCTTTCG	CACAGCACGTTGATTGCACC
Slc39a1	AGGTCAGGTGCTAACCATGAA	CTGTTCCTTGTAAGCCAGCGT
Slc39a14	GAGTGGGCCGGGATAATGTTT	GAGATCGCTCGCTCAAGTTGT
Slc39a4	ATGCTCCCAAAGTCGGTCAC	CAGCGTATTTAACAGGCCGTC
Srebp1	TGACCCGGCTATTCCGTGA	CTGGGCTGAGCAATACAGTTC
Tnfa	CCCTCACACTCAGATCATCTTCT	GCTACGACGTGGGCTACAG
Ugt1a1	GCTTCTTCCGTACCTTCTGTTG	GCTGCTGAATAACTCCAAGCAT
Ugt1a6a	GTTTCTCTTCCTAGTGCTTTGGG	CCTCGTTCACTGAGATGTTCTAC
Ugt1a6b	TACAGGAACAGCATGCTTACAT	GTTGAGA TACTCAGCCAGGAT
